# Supplementary material for: Unravelling reference bias in ancient DNA datasets
Source: Bioinformatics. 2024 Jul 3;40(7):btae436. doi: 10.1093/bioinformatics/btae436 (PMC11254355; doi:10.1093/bioinformatics/btae436)
Supplement: btae436_Supplementary_Data [file btae436_supplementary_data.zip › S1-S4_data_plots.zip!/S1-S4_data/elephant_sim_plots.zip!/elephant_sim_plots/bwamem_elephant_div15_1end_by_MQ.pdf]

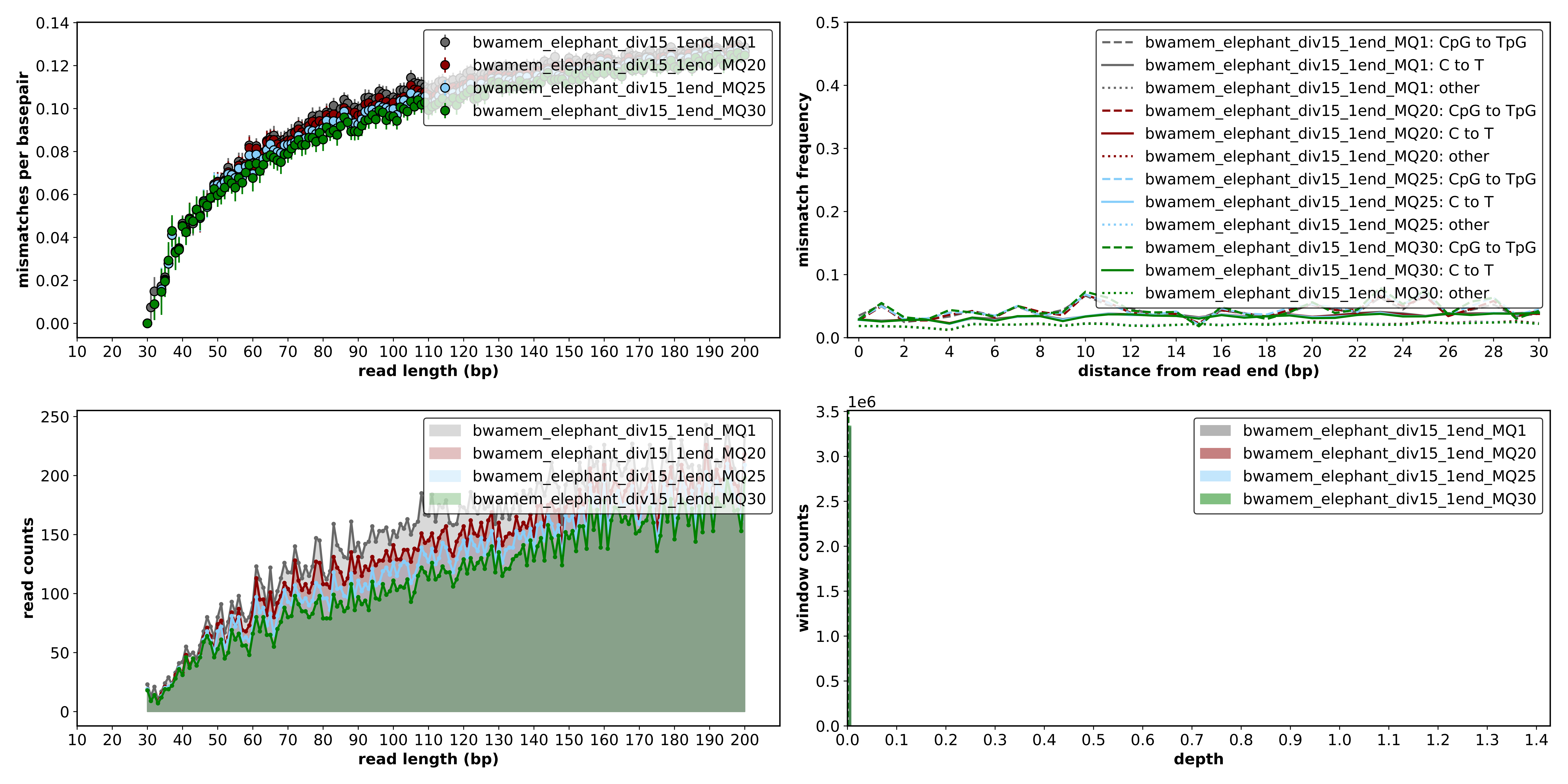

mismatches per basepair
read length (bp)
bwamem_elephant_div15_1end_MQ1
bwamem_elephant_div15_1end_MQ20
bwamem_elephant_div15_1end_MQ25
bwamem_elephant_div15_1end_MQ30
0.00
0.02
0.04
0.06
0.08
0.10
0.12
0.14
10 20 30 40 50 60 70 80 90 100 110 120 130 140 150 160 170 180 190 200
mismatch frequency
distance from read end (bp)
bwamem_elephant_div15_1end_MQ1: CpG to TpG
bwamem_elephant_div15_1end_MQ1: C to T
bwamem_elephant_div15_1end_MQ1: other
bwamem_elephant_div15_1end_MQ20: CpG to TpG
bwamem_elephant_div15_1end_MQ20: C to T
bwamem_elephant_div15_1end_MQ20: other
bwamem_elephant_div15_1end_MQ25: CpG to TpG
bwamem_elephant_div15_1end_MQ25: C to T
bwamem_elephant_div15_1end_MQ25: other
bwamem_elephant_div15_1end_MQ30: CpG to TpG
bwamem_elephant_div15_1end_MQ30: C to T
bwamem_elephant_div15_1end_MQ30: other
0.0
0.1
0.2
0.3
0.4
0.5
0 2 4 6 8 10 12 14 16 18 20 22 24 26 28 30
read counts
read length (bp)
bwamem_elephant_div15_1end_MQ1
bwamem_elephant_div15_1end_MQ20
bwamem_elephant_div15_1end_MQ25
bwamem_elephant_div15_1end_MQ30
0
50
100
150
200
250
10 20 30 40 50 60 70 80 90 100 110 120 130 140 150 160 170 180 190 200
window counts
depth
1e6
bwamem_elephant_div15_1end_MQ1
bwamem_elephant_div15_1end_MQ20
bwamem_elephant_div15_1end_MQ25
bwamem_elephant_div15_1end_MQ30
0.0
0.5
1.0
1.5
2.0
2.5
3.0
3.5
0.0 0.1 0.2 0.3 0.4 0.5 0.6 0.7 0.8 0.9 1.0 1.1 1.2 1.3 1.4
